# Cytokine patterns in very low birth weight infants under different cord clamping strategies: preliminary EXPLAIN trial data

**DOI:** 10.1186/s12887-026-07097-7

**Published:** 2026-06-08

**Authors:** Benjamin Kuehne, Mohamed Majjouti, Jochen Wilhelm, Sarina K. Butzer, Angela Kribs, Miguel A. Alejandre Alcazar, Esther Mahabir, André Oberthuer

**Affiliations:** 1 Department of Pediatrics, Division of Neonatology, University of Cologne, Faculty of Medicine and University Hospital Cologne, Kerpener Str. 62, Cologne, 50937 Germany; 2 Comparative Medicine, Center for Molecular Medicine Cologne, University of Cologne, Faculty of Medicine and University Hospital Cologne, Cologne, Germany; 3 Current address: Testo Industrial Services GmbH, Gewerbestraße 3, Kirchzarten, 79199 Germany; 4 Institute for Lung Health (ILH), University of Giessen and Marburg Lung Center (UGMLC) and Cardiopulmonary Institute (CPI), Member of the German Center for Lung Research (DZL), Justus-Liebig University, Giessen, Germany; 5 Department of Pediatrics, Translational Experimental Pediatrics, University of Cologne, Faculty of Medicine and University Hospital Cologne, Cologne, Germany; 6 Center for Molecular Medicine Cologne (CMMC) and Cologne Excellence Cluster On Cellular Stress Responses in Aging-Associated Diseases (CECAD), University of Cologne, Faculty of Medicine and University Hospital Cologne, Cologne, Germany; 7 Comparative Medicine, Center for Molecular Medicine Cologne, University of Cologne, Faculty of Medicine and University Hospital Cologne, Cologne, Germany

**Keywords:** Umbilical cord, Very low birth weight infant, Cytokines, Extrauterine placental perfusion, Neonatal transition, Delayed cord clamping

## Abstract

**Background:**

Delayed umbilical cord clamping (DCC) improves survival and hematologic outcomes in preterm infants. Strategies that provide initial respiratory support before cord clamping may prolong intact placental circulation beyond standard time-based DCC in very low birth weight (VLBW) infants. Whether prolonged intact placental circulation modifies neonatal cytokine patterns remains unclear.

**Methods:**

This pre-specified secondary analysis of a single-center randomized controlled trial (Extrauterine Placental Transfusion in Resuscitation of VLBW Infants [EXPLAIN]) included preterm infants with a birth weight < 1500 g and gestational age > 23 6/7 weeks delivered by cesarean section between May 2019 and June 2021. Infants were randomized to either extrauterine placental perfusion (EPP group) with intact placental circulation or time-based DCC (DCC group). Samples were obtained from umbilical cord blood at cord clamping (d0) and venous blood on postnatal day 28 (d28). Twenty-seven cytokines were quantified using multiplex ELISAs.

**Results:**

Samples were available from 44 infants; 1 infant was excluded a priori because of neonatal sepsis, leaving 43 infants in the cytokine analysis set. Mean time until cord clamping was 469.6 s in the EPP group and 40.8 s in the DCC group (*p* < 0.001). At d0, significant relative differences between groups were observed for IL-1ra, IL-17, and IP-10. At d28, EPP was associated with lower IP-10 concentrations than DCC. No broad or consistent differences in cytokine profiles were observed between groups.

**Conclusions:**

In this small exploratory secondary analysis, EPP was not associated with broad cytokine changes compared with DCC in VLBW infants, despite markedly prolonged placental circulation. These findings may support the biological feasibility of EPP and may indicate that prolonged placental perfusion is not associated with major systemic cytokine perturbation. Given the limited sample size, the results should be considered hypothesis-generating and require confirmation in larger studies.

**Trial registration:**

Clinicaltrials.gov (NCT03916159, registered April 05, 2019).

**Supplementary Information:**

The online version contains supplementary material available at 10.1186/s12887-026-07097-7.

## Background

Delayed umbilical cord clamping (DCC) is widely practiced to support neonatal transition in preterm infants and is associated with lower mortality before discharge and improved hematologic outcomes compared with immediate cord clamping (ICC). Although the optimal duration of DCC is still being defined, longer delays (≥ 120 s) may confer greater survival benefit in preterm infants, while shorter intervals (30–60 s) remain beneficial and are commonly applied in clinical trials [1, 2]. While these clinical benefits are increasingly recognized, the extent to which different cord management strategies influence neonatal immune and inflammatory signaling remains poorly understood.

The proposed mechanism underlying these benefits is improved cardiopulmonary transition, facilitated by ongoing placental perfusion augmented by lung aeration. Initial breaths enhance placental transfusion, supporting strategies that integrate respiratory support before umbilical cord clamping. Most very low birth weight (VLBW) infants require continuous-positive-airway-pressure (CPAP) respiratory support to achieve adequate lung aeration during neonatal transition. Maintaining intact placental circulation during this period can prolong placental perfusion compared to standard time-based DCC of 30–60 s [3]. While this extended period may maximize placental transfusion, it also lengthens the circulation of umbilical cord blood in the neonate, potentially altering neonatal exposure to soluble mediators such as cytokines, which may influence inflammation, immune maturation, or postnatal outcomes. Nevertheless, approaches that maintain intact placental circulation with an intact umbilical cord have been associated with improved stabilization and oxygenation in preterm infants [4].

Despite growing evidence for hemodynamic and hematologic benefits of DCC and intact-umbilical-cord approaches, data on inflammatory signaling are sparse [5]. Proinflammatory cytokines are elevated in cord blood of term and preterm infants [6], suggesting a physiological role in parturition independent of chorioamnionitis [7]. However, the extent to which different cord management strategies influence cytokine transfer and postnatal cytokine profiles is still unknown, particularly in VLBW infants [6, 8]. It remains unclear whether prolonged intact placental circulation during stabilization alters early systemic inflammatory signaling through differences in placental-fetal exchange, oxygenation, and transitional physiology.

We hypothesized that extended placental perfusion in VLBW infants would modify neonatal cytokine profiles compared with shorter time-based DCC. The present study was not designed to establish reference cytokine ranges for preterm infants. Rather, it represents a pre-specified exploratory secondary analysis of the *Extrauterine Placental Transfusion in Resuscitation of VLBW Infants* (EXPLAIN) trial. In this secondary analysis, we evaluated 27 cytokines in serum from umbilical cord blood after cord clamping and from neonates on day 28 to address this gap.

## Methods

### Study design and reporting compliance

The study was a pre-specified secondary analysis of the EXPLAIN trial (NCT03916159), a single-center randomized controlled trial conducted at the University Hospital of Cologne, Germany, between May 2019 and June 2021. Infants enrolled in the EXPLAIN trial were eligible for the cytokine analysis. The trial included infants born by cesarean section (CS) with birthweight < 1500 g and gestational age > 23 6/7 weeks and assigned them to either extrauterine placental perfusion (EPP group; delivery with maintained intact placental circulation and respiratory support with an intact umbilical cord) or time-based delayed cord clamping (DCC group). Participants were randomized 1:1 using a computer-generated sequence with variable block sizes. Masking of delivery-room clinicians was not feasible; laboratory personnel were blinded to group assignment. The study was conducted and reported in accordance with the CONSORT principles; operational details are provided in the main study protocol [4].

### Type of sampling and reasons for selection

For the present analysis, infants were included if biological samples for cytokine measurements were available. Cytokine analyses were based on umbilical cord blood collected immediately after cord clamping (d0) and venous blood obtained on postnatal day 28 (± 2 days), whenever feasible. These time points were chosen to assess cytokine patterns both during immediate postnatal transition and at the end of the neonatal period.

### Ethical considerations

The trial was approved by the local ethics committee of the University of Cologne and conducted in accordance with the Declaration of Helsinki and the International Conference on Harmonization's guidelines for good clinical practice.

### Inclusion and exclusion criteria

Infants in the extrauterine placental perfusion (EPP) group were born with the placenta still attached and resuscitated with continuous-positive-airway-pressure (CPAP) respiratory support with intact umbilical cord. Umbilical cord clamping in these infants was delayed for at least 60 s and could be prolonged for several minutes. Infants in the DCC group received time-based cord clamping for at least 30 s, after which the umbilical cord was clamped, and CPAP was initiated post-clamping.

Exclusion criteria for the primary study included vaginal delivery, fetal or maternal risk (i.e., compromise, emergency CS and/or general anesthesia), placental abruption or placenta previa with hemorrhage, placenta accreta or increta, monochorionic multiples, congenital anomalies, and lack of prenatal parental consent.

### Patient consent statement

Written prenatal informed consent was obtained from the parents or legal guardians of all included infants before enrollment in the primary trial.

### Maternal and neonatal data

All study data were collected from infant and mother medical records and managed using REDCap electronic data capture tools [9]. Intrauterine growth restriction (IUGR) was defined as less than the 10th percentile for both weight and length at birth. Clinical chorioamnionitis was defined on the basis of maternal intrapartum clinical findings, namely maternal fever of at least 100 °F (37.8 °C) and/or maternal serum C-reactive protein (CRP) > 2.0 mg/dL, as used in the local clinical setting [10–12]. Bronchopulmonary dysplasia (BPD) was categorized according to the definition of Walsh et al. [13]. Intraventricular hemorrhage (IVH) was staged according to the criteria of Papile et al. [14].

### Blood sample collection and estimation of cytokines

Immediately after umbilical cord clamping in both groups, blood samples (1–2 ml) were collected from the umbilical vein. Venous blood samples (0.5 ml) were collected on the 28th (± 2 days) day after birth as part of a routine blood sampling. Samples were kept in the blood collection tubes (Sarstedt AG & Co. KG, Germany) for 30 min after collection and then centrifuged for 5 min at room temperature. The serum was then aliquoted and stored in storage tubes at −80 °C until analysis.

In total, 27 cytokines were measured in duplicate via enzyme-linked immunosorbent assay (ELISA) in a multiplex analyzer (Bio-Plex 200®, Bio-Rad Laboratories, USA) according to the manufacturer’s instructions. These cytokines were interleukin (IL)−1β, IL-1 receptor antagonist (ra), IL-2, IL-4, IL-5, IL-6, IL-7, IL-8, IL-9, IL-10, IL-12 (p70), IL-13, IL-15, IL-17, eotaxin, basic fibroblast growth factor (FGF basic), granulocyte colony-stimulating factor (G-CSF), granulocyte/macrophage colony-stimulating factor (GM-CSF), interferon-γ (IFN-γ), IFN-γ-induced protein 10 (IP-10), monocyte chemotactic protein-1 (MCP-1), macrophage inflammatory protein-1α (MIP-1α), MIP-1β, platelet-derived growth factor-bb (PDGF-bb), “Regulated on Activation, Normal T-cell Expressed and Secreted” (RANTES), tumor necrosis factor-α (TNF-α), and vascular endothelial growth factor (VEGF) (Bio-Rad Laboratories). Sera were thawed, centrifuged for 10 min at 10,000 g and 4 °C and diluted in sample diluent (1:4). By using the median of the fluorescence intensity and the standard curve, the absolute concentration of the cytokines (pg/mL) was calculated (Bio-Plex Manager 6.1, Bio-Rad Laboratories).

### Statistical analysis

Descriptive statistics and baseline comparisons: Distributions of variables are summarized as mean and standard deviation (SD), as median and interquartile range (IQR) or absolute and relative frequencies. Differences between groups were compared by Mann–Whitney U-test (ordinal outcomes) or Fisher’s exact test (nominal outcomes). A two-tailed *p*-value < 0.05 was considered statistically significant.

Modeling of cytokines: Logarithms of cytokine concentrations were analyzed depending on intervention (clamping method, DCC or EPP) and time (d0 or d28) using left-censored mixed linear models. Values below the lower limit of quantification (LLOQ)(Table 2) were treated as left-censored at the cytokine-specific LLOQ. The models were fit in R (version 3.5.1, [15]) using the function *survreg* from the survival package (version 3.6–4, [16]) to analyze simple effects of the intervention within the two time points and using the function lmercens from the package lm4cens (version 0.1.14, [17]) to evaluate the intervention-time interaction. In cases without censoring, these models were fit using the function lmer from the package lmerTest (version 3.1–3). Primary models were adjusted for antenatal corticosteroid exposure (any vs none) and preeclampsia (yes/no). Effects are reported as relative differences (RD) with 95% confidence intervals (CI). RD > 1 indicates higher concentrations in DCC relative to EPP.

## Results

**Fig. 1:**
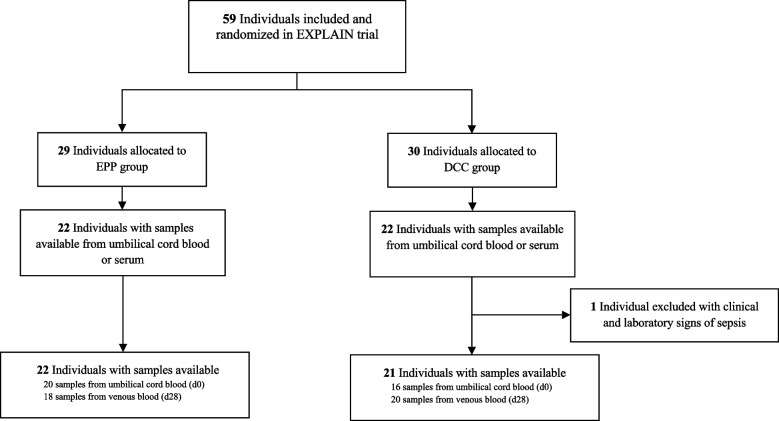
Study flowchart. Abbreviations: DCC, delayed cord clamping; EPP, extrauterine placental perfusion

In the original EXPLAIN trial, 59 infants were randomized including 29 infants in the EPP group and 30 infants in the DCC group. For the present cytokine analysis, infants were eligible if samples for cytokine measurements were available. Samples were obtained, when feasible, from umbilical cord blood at birth (d0) and venous blood at 28 days after birth (d28). Of 44 infants with available samples, one infant was excluded a priori because of clinical and laboratory signs of neonatal sepsis, leaving 43 infants in the cytokine analysis set. Overall, 22 infants in the EPP group and 21 infants in the DCC group contributed samples for cytokine analyses (Fig. 1).

Baseline maternal and neonatal characteristics and short-term outcomes of the infants included in the analysis are shown in Table 1. Baseline characteristics were largely comparable between groups; however, antenatal steroid exposure was more frequent in the EPP group than in the DCC group (100% vs. 81.0%, *p* = 0.048) whereas preeclampsia was more common in the DCC group than in the EPP group (38.1% vs. 4.5%, *p* = 0.01). These variables were included as covariates in the adjusted cytokine analyses. The mean time until umbilical cord clamping was 469.6 (± 179.0) seconds in the EPP group and 40.8 (± 8.8) seconds in the DCC group (median [IQR]: 475 [400–580] seconds vs 40 [31–45] seconds, respectively, *p* < 0.001) (Table 1).

**Table 1:** Infant and maternal demographics and outcomes

Parameter	All (*n* = 43)	EPP (*n* = 22)	DCC (*n* = 21)	*P*-value^a^
Gestational age, mean (wk + days) (SD) (days)	28 + 1 (16.3)	27 + 6 (15.0)	28 + 4 (17.7)	0.42
Birth weight, mean (SD), g	979.1 (313.0)	975.2 (312.1)	983.1 (321.5)	0.98
Sex				0.54
• Female, No. (%)	24 (55.8)	11 (50.0)	13 (61.9)	N/A
• Male, No. (%)	19 (44.2)	11 (50.0)	8 (38.1)	N/A
Multiple birth, No. (%)	6 (14.0)	2 (9.1)	4 (19.0)	0.41
Primigravida, No. (%)	23 (53.5)	12 (54.5)	11 (52.4)	0.88
Maternal age, median [IQR]	35 [30–37]	33 [29–37]	35 [32–36]	0.31
Antenatal corticosteroids, No. (%)	39 (92.9)	22 (100)	17 (81.0)	0.048*
• Partial course, No. (%)	8 (18.6)	3 (13.6)	5 (23.8)	N/A
• Full course, No. (%)	31 (72.1)	19 (86.4)	12 (57.1)	N/A
PROM, No. (%)	10 (23.3)	6 (27.3)	4 (19.1)	0.72
• PROM > 24 h, No. (%)	8 (18.6)	6 (27.3)	2 (9.5)	0.24
Clinical chorioamnionitis, No. (%)	2 (4.7)	2 (9.1)	0 (0)	0.49
Preeclampsia, No. (%)	9 (20.9)	1 (4.5)	8 (38.1)	0.01*
HELLP, No. (%)	2 (4.7)	1 (4.5)	1 (4.8)	> 0.99
IUGR, No. (%)	13 (30.2)	4 (18.2)	9 (42.9)	0.10
Active labour, No. (%)	12 (27.9)	6 (27.3)	6 (28.6)	> 0.99
Apgar score, median [IQR]				
• 5 min	8 [7, 8]	7 [7, 8]	8 [7, 8]	0.45
• 10 min	9 [8, 9]	8 [8, 9]	9 [8, 9]	0.12
Time until umbilical cord clamping, mean (SD), median [IQR], sec	N/A	469.6 (179.0) 475 [400–580]	40.8 (8.8) 40 [31–45]	< 0.001*
Received surfactant in delivery room, No. (%)	41 (95.3)	22 (100)	19 (90.5)	0.23
Infant blood transfusion during first 7 days of life, No. (%)	2 (4.7)	1 (4.5)	1 (4.8)	> 0.99
Mechanical ventilation, No. (%)	11 (25.6)	7 (31.8)	4 (19.1)	0.49
Duration of mechanical ventilation, median [IQR], h	255 [167–495]	255 [167–912]	251 [140–437]	0.93
Pneumothorax, No. (%)	3 (7.0)	1 (4.5)	2 (9.5)	0.61
Bronchopulmonary dysplasia at 36 weeks corrected				0.46
• Total with data, No	40	19	21	N/A
• mild, No. (%)	20 (50.0)	11 (58.0)	9 (43.0)	N/A
• moderate, No. (%)	2 (5.0)	1 (5.3)	1 (4.8)	N/A
• severe, No. (%)	1 (2.5)	1 (5.3)	0 (0)	N/A
Necrotizing enterocolitis with surgery, No (%)	2 (4.7)	2 (9.1)	0 (0)	0.49
Spontaneous intestinal perforation with surgery, No. (%)	4 (9.3)	2 (9.1)	2 (9.5)	> 0.99
Intraventricular hemorrhage	9 (20.9)	6 (27.3)	3 (14.3)	0.52
• Grade 1, No (%)	7 (16.3)	4 (18.2)	3 (14.3)	N/A
• Grade 2, No (%)	1 (2.5)	1 (4.5)	0 (0)	N/A
• Grade ≥ 3, No (%)	1 (2.5)	1 (4.5)	0 (0)	N/A
Periventricular Leukomalacia				
• Total with data, No	42	21	21	N/A
• Yes	0 (0)	0 (0)	0 (0)	N/A
Retinopathy necessitating intervention (medical/surgical)				
• Total with data, No	42	21	20	N/A
• Yes	2 (4.8)	1 (4.8)	1 (5.0)	> 0.99
Survival until discharge, No. (%)	42 (97.7)	21 (95.5)	21 (100)	> 0.99

*Abbreviations*: *EPP* Extrauterine placental perfusion, *DCC* Delayed cord clamping, *h* hours, *HELLP* Hemolysis, elevated liver enzymes, and low platelet count, *IQR* Interquartile range, *IUGR* Intrauterine growth restriction, *N/A* Not applicable, *PROM* Prolonged rupture of membranes, *SD* Standard deviation

^a^*p*-value is based on Fisher’s exact test for categorial variables and Mann–Whitney-U test for continuous variables

^*^p ≤ 0.05 indicates statistical significance

Overall, a maximum of 36 samples at d0 and 38 samples at d28 were available for analysis. The maximum number of samples available per group was 20 (EPP group) and 16 (DCC group) at d0, and 18 (EPP group) and 20 (DCC group) at d28 (Fig. 1). Because the number of available and quantifiable samples varied across cytokines, the exact denominator for each analyte and time point is reported in Table 2. The distribution of concentrations, LLOQ and ULOQ (upper limit of quantification) is also shown in Table 3. None of the analyzed samples had cytokine concentrations above the ULOQ. A high proportion of samples showed values below the LLOQ for several cytokines including IL-2, IL-5, IL-7, IL-10, IL-13, IL-15, GM-CSF, and VEGF (Table 3).

**Table 2:** Mean measurable concentrations of cytokines at d0 and d28 compared by sub-groups and over time

	**Measurable range EPP group, n (%)**	**EPP group, mean** ± **SD, pg/ml**	**Measurable range DCC group, n (%)**	**DCC group, mean** ± **SD, pg/ml**	***p*-value**
*Pro-inflammatory cytokines*					
TNFα, d0	20 (100.0)	89.37 ± 20.26	16 (100.0)	87.77 ± 18.20	0.66
TNFα, d28	18 (100.0)	86.68 ± 23.02	20 (100.0)	96.56 ± 20.20	0.11
IL-1β, d0	20 (100.0)	2.18 ± 0.89	16 (100.0)	2.63 ± 2.47	0.64
IL-1β, d28	18 (100.0)	3.02 ± 1.24	20 (100.0)	3.46 ± 1.79	0.67
IL-6, d0	18 (90.0)	17.12 ± 20.07	15 (93.8)	8.39 ± 10.51	0.91
IL-6, d28	13 (72.2)	8.48 ± 20.24	17 (85.0)	4.90 ± 6.35	0.37
*Anti-inflammatory cytokines*					
IL-1ra, d0	20 (100.0)	863.51 ± 1,091.38	15 (93.8)	392.46 ± 295.04	0.03*
IL-1ra, d28	17 (85.0)	2,012.78 ± 2,885.00	20 (100.0)	2,669.60 ± 4,661.53	0.24
***Th1 cytokines***					
IFNγ, d0	20 (100.0)	14.52 ± 13.77	16 (100.0)	12.81 ± 15.62	0.58
IFNγ, d28	18 (100.0)	17.87 ± 16.14	20 (100.0)	24.22 ± 32.78	0.41
IL-2, d0	9 (45.0)	3.05 ± 2.22	10 (62.5)	4.57 ± 6.12	1.00
IL-2, d28	8 (44.4)	2.42 ± 2.98	12 (60.0)	4.24 ± 4.80	0.24
IL-12 (p70), d0	14 (70.0)	7.52 ± 11.19	12 (75.0)	8.12 ± 10.24	0.07
IL-12 (p70), d28	13 (72.2)	2.88 ± 1.83	11 (55.0)	4.50 ± 3.10	1.00
IL-15, d0	3 (15.0)	175.98 ± 124.35	3 (18.8)	202.40 ± 24.42	1.00
IL-15, d28	1 (5.6)	79.70 ± 0.00	2 (10.0)	228.20 ± 74.25	N/A
*Th17 cytokine*					
IL-17, d0	20 (100.0)	7.62 ± 2.26	16 (100.0)	9.27 ± 6.60	< 0.001*
IL-17, d28	18 (100.0)	15.20 ± 6.36	20 (100.0)	16.21 ± 5.91	0.87
*Th2 cytokines*					
IL-4, d0	20 (100.0)	2.00 ± 0.99	16 (100.0)	2.22 ± 1.44	0.74
IL-4, d28	18 (100.0)	5.11 ± 2.69	20 (100.0)	5.31 ± 2.18	0.91
IL-5, d0	8 (40.0)	42.20 ± 32.43	12 (75.0)	22.04 ± 18.15	0.43
IL-5, d28	7 (38.9)	17.74 ± 22.45	10 (50.0)	27.49 ± 19.74	0.16
IL-9, d0	20 (100.0)	340.99 ± 35.41	16 (100.0)	343.83 ± 53.65	0.64
IL-9, d28	18 (100.0)	361.17 ± 54.39	20 (100.0)	370.06 ± 35.47	0.98
IL-10, d0	5 (25.0)	3.90 ± 2.67	5 (31.3)	2.34 ± 1.79	1.00
IL-10, d28	1 (5.6)	8.48 ± 0.00	4 (20.0)	19.99 ± 21.09	0.07
IL-13, d0	17 (85.0)	0.53 ± 0.46	16 (100.0)	0.65 ± 1.02	0.09
IL-13, d28	17 (95.4)	0.94 ± 0.56	19 (95.0)	1.05 ± 0.54	0.51
*Growth factors*					
FGF-basic, d0	20 (100.0)	54.63 ± 15.49	16 (100.0)	63.17 ± 38.87	0.43
FGF-basic, d28	18 (100.0)	54.90 ± 15.21	20 (100.0)	55.90 ± 16.57	0.68
IL-7, d0	13 (65.0)	13.69 ± 26.10	10 (62.5)	11.22 ± 12.08	0.56
IL-7, d28	15 (83.3)	12.64 ± 7.77	18 (90.0)	13.03 ± 8.26	0.51
GM-CSF, d0	12 (60.0)	1.64 ± 1.17	10 (62.5)	2.81 ± 2.45	0.57
GM-CSF, d28	12 (66.7)	1.99 ± 1.48	15 (75.0)	2.25 ± 1.95	0.52
G-CSF, d0	20 (100.0)	129.83 ± 68.92	16 (100.0)	314.39 ± 730.71	0.34
G-CSF, d28	18 (100.0)	159.12 ± 126.43	20 (100.0)	284.59 ± 541.21	0.27
PDGF-BB, d0	20 (100.0)	573.75 ± 578.23	16 (100.0)	837.36 ± 597.09	0.09
PDGF-BB, d28	16 (100.0)	800.62 ± 1,014.57	19 (95.0)	861.52 ± 1,000.66	0.62
VEGF, d0	5 (25.0)	173.85 ± 102.78	5 (31.3)	78.94 ± 41.28	0.24
VEGF, d28	1 (5.6)	257.60 ± 0.00	3 (15.0)	136.59 ± 73.79	0.28
*Chemokines*					
Eotaxin, d0	20 (100.0)	17.76 ± 12.62	16 (100.0)	18.42 ± 13.46	0.54
Eotaxin, d28	18 (100.0)	52.83 ± 35.46	20 (100.0)	57.62 ± 27.31	0.54
IL-8, d0	20 (100.0)	41.83 ± 31.94	16 (100.0)	36.72 ± 29.82	0.47
IL-8, d28	18 (100.0)	40.16 ± 55.04	20 (100.0)	43.81 ± 103.53	0.63
IP-10, d0	20 (100.0)	339.85 ± 354.87	16 (100.0)	184.05 ± 119.89	0.04*
IP-10, d28	18 (100.0)	210.96 ± 123.44	20 (100.0)	877.27 ± 2,336.78	0.004*
MCP-1, d0	20 (100.0)	83.89 ± 76.91	16 (100.0)	66.94 ± 46.41	0.88
MCP-1, d28	18 (100.0)	56.47 ± 52.60	20 (100.0)	76.29 ± 84.56	0.39
MIP-1α, d0	20 (100.0)	5.16 ± 3.42	16 (100.0)	101.88 ± 387.19	0.99
MIP-1α, d28	18 (100.0)	5.57 ± 7.31	20 (100.0)	25.50 ± 91.23	0.33
MIP-1β, d0	20 (100.0)	246.33 ± 27.00	16 (100.0)	243.82 ± 40.80	0.62
MIP-1β, d28	18 (100.0)	251.95 ± 30.54	20 (100.0)	271.87 ± 31.87	0.10
RANTES, d0	20 (100.0)	12,985.60 ± 4,514.43	16 (100.0)	12,697.81 ± 4,977.47	0.43
RANTES, d28	18 (100.0)	9,766.78 ± 4,743.73	20 (100.0)	11,735.66 ± 4,333.78	0.20

Summary statistics according to the number of infants with detectable cytokine levels

*Abbreviations*: *EPP* Extrauterine placental perfusion, *DCC* Delayed cord clamping, *SD* Standard deviation

^*^p ≤ 0.05 indicates statistical significance

**Table 3:** Distribution of concentrations of the 27 cytokines

	Measurable range, n (%)	LLOQ (pg/ml)	< LLOQ, n (%)	< 1.0 pg/ml, n (%)	> 100.0 pg/ml, n (%)	ULOQ (pg/ml)	> ULOQ, n (%)
Pro-inflammatory cytokines							
TNFα, d0	36 (100)	3.35	0 (0)	0 (0)	8 (22.2)	54,925	0 (0)
TNFα, d28	38 (100)	3.35	0 (0)	0 (0)	8 (22.2)	54,925	0 (0)
IL-1β, d0	32 (84.2)	0.27	0 (0)	1 (3.1)	0 (0)	4,467	0 (0)
IL-1β, d28	38 (100)	0.27	0 (0)	0 (0)	0 (0)	4,467	0 (0)
IL-6, d0	32 (84.2)	0.42	3 (8.6)	3 (8.6)	0 (0)	6,918	0 (0)
IL-6, d28	16 (42.1)	0.42	8 (33.3)	8 (33.3)	0 (0)	6,918	0 (0)
Anti-inflammatory cytokines							
IL-1ra, d0	34 (94.4)	10.76	1 (2.9)	0 (0)	33 (97.1)	176,363	0 (0)
IL-1ra, d28	36 (94.7)	10.76	1 (2.7)	0 (0)	34 (94.4)	176,363	0 (0)
Th1 cytokines							
IFNγ, d0	35 (97.2)	0.6	0 (0)	0 (0)	0 (0)	9,820	0 (0)
IFNγ, d28	38 (100)	0.6	0 (0)	0 (0)	1 (2.6)	9,820	0 (0)
IL-2, d0	1 (2.8)	1.7	18 (94.7)	0 (0)	0 (0)	27,811	0 (0)
IL-2, d28	4 (10.5)	1.7	24 (85.7)	0 (0)	0 (0)	27,811	0 (0)
IL-12 (p70), d0	5 (13.9)	1.87	10 (66.7)	0 (0)	0 (0)	30,667	0 (0)
IL-12 (p70), d28	2 (5.3)	1.87	14 (87.5)	0 (0)	0 (0)	30,667	0 (0)
IL-15, d0	1 (2.8)	18.02	30 (96.8)	0 (0)	1 (100)	295,216	0 (0)
IL-15, d28	0 (0)	18.02	35 (100)	0 (0)	0 (0)	295,216	0 (0)
Th2 cytokines							
IL-4, d0	35 (97.2)	0.21	0 (0)	1 (2.9)	0 (0)	3,422	0 (0)
IL-4, d28	38 (100)	0.21	0 (0)	0 (0)	0 (0)	3,422	0 (0)
IL-5, d0	10 (38.5)	5.34	16 (61.5)	0 (0)	1 (3.9)	87,491	0 (0)
IL-5, d28	6 (22.2)	5.34	21 (77.8)	0 (0)	0 (0)	87,491	0 (0)
IL-9, d0	36 (100)	1.23	0 (0)	0 (0)	36 (100)	20,125	0 (0)
IL-9, d28	38 (100)	1.23	0 (0)	0 (0)	38 (100)	20,125	0 (0)
IL-10, d0	4 (13.3)	0.81	26 (86.7)	26 (86.7)	0 (0)	13,284	0 (0)
IL-10, d28	5 (13.2)	0.81	33 (86.8)	33 (86.8)	0 (0)	13,284	0 (0)
IL-13, d0	2 (40.0)	0.34	3 (60.0)	3 (60.0)	0 (0)	5,599	0 (0)
IL-13, d28	12 (85.7)	0.34	2 (14.3)	2 (14.3)	0 (0)	5,599	0 (0)
Th17 cytokine							
IL-17, d0	6 (16.7)	2.67	0 (0)	0 (0)	0 (0)	43,802	0 (0)
IL-17, d28	31 (82.0)	2.67	0 (0)	0 (0)	0 (0)	43,802	0 (0)
Growth factors							
FGF-basic, d0	36 (100)	3.66	0 (0)	0 (0)	0 (0)	59,965	0 (0)
FGF-basic, d28	38 (100)	3.66	0 (0)	0 (0)	0 (0)	59,965	0 (0)
IL-7, d0	9 (37.5)	2.6	15 (62.5)	0 (0)	0 (0)	42,680	0 (0)
IL-7, d28	22 (81.5)	2.6	5 (18.5)	0 (0)	0 (0)	42,680	0 (0)
GM-CSF, d0	11 (44.0)	0.44	14 (56.0)	14 (56.0)	0 (0)	7,265	0 (0)
GM-CSF, d28	14 (56.0)	0.44	11 (44.0)	11 (44.0)	0 (0)	7,265	0 (0)
G-CSF, d0	36 (100)	4.34	0 (0)	0 (0)	25 (69.4)	71,162	0 (0)
G-CSF, d28	38 (100)	4.34	0 (0)	0 (0)	26 (68.4)	71,162	0 (0)
PDGF-BB, d0	35 (100)	2.46	0 (0)	0 (0)	33 (94.3)	40,349	0 (0)
PDGF-BB, d28	35 (92.1)	2.46	3 (8.6)	0 (0)	31 (88.6)	40,349	0 (0)
VEGF, d0	7 (21.2)	4.22	26 (78.8)	0 (0)	6 (85.7)	69,212	0 (0)
VEGF, d28	4 (10.3)	4.22	35 (89.7)	0 (0)	2 (50.0)	69,212	0 (0)
Chemokines							
Eotaxin, d0	36 (100)	0.09	0 (0)	0 (0)	0 (0)	1,488	0 (0)
Eotaxin, d28	38 (100)	0.09	0 (0)	0 (0)	4 (0)	1,488	0 (0)
IL-8, d0	36 (100)	0.81	0 (0)	0 (0)	3 (8.3)	13,259	0 (0)
IL-8, d28	38 (100)	0.81	0 (0)	0 (0)	3 (7.9)	13,259	0 (0)
IP-10, d0	36 (100)	1.41	0 (0)	0 (0)	29 (80.6)	23,159	0 (0)
IP-10, d28	38 (100)	1.41	0 (0)	0 (0)	35 (92.1)	23,159	0 (0)
MCP-1, d0	36 (100)	0.58	0 (0)	0 (0)	9 (25.0)	9,569	0 (0)
MCP-1, d28	38 (100)	0.58	0 (0)	0 (0)	7 (18.4)	9,569	0 (0)
MIP-1α, d0	35 (100)	0.09	0 (0)	0 (0)	0 (0)	1,461	0 (0)
MIP-1α, d28	37 (100)	0.09	0 (0)	2 (5.4)	0 (0)	1,461	0 (0)
MIP-1β, d0	36 (100)	0.36	0 (0)	0 (0)	36 (100)	5,906	0 (0)
MIP-1β, d28	38 (100)	0.36	0 (0)	0 (0)	38 (100)	5,906	0 (0)
RANTES, d0	35 (100)	1.37	0 (0)	0 (0)	35 (100)	22,379	0 (0)
RANTES, d28	36 (100)	1.37	0 (0)	0 (0)	36 (100)	22,379	0 (0)

For each cytokine, n refers to the number of samples with available measurements at the respective time point. Percentages for measurable range, values below the LLOQ, values < 1.0 pg/ml, values > 100.0 pg/ml, and values above the ULOQ were calculated using the cytokine-specific denominator at that time point

*Abbreviations*: *LLOQ* Lower limit of quantification, *N/A* Not applicable, *ULOQ* Upper limit of quantification

Adjusted cytokine estimates were derived from left-censored mixed linear models, with relative differences (RD) > 1 indicating higher cytokine concentrations in DCC relative to EPP. At d0, the adjusted mean cytokine concentrations were significantly lower for IL-1ra (RD, 95% confidence interval [CI]: 0.19–0.91) and IP-10 (RD, 95% CI: 0.27–0.98) in the DCC group than in the EPP group. Descriptively, mean IL-1ra concentrations at d0 were 863.51 ± 1,091.38 pg/mL in the EPP group and 392.46 ± 295.04 pg/mL in the DCC group, and mean IP-10 concentrations were 339.85 ± 354.87 pg/mL in the EPP group and 184.05 ± 119.89 pg/mL in the DCC group, respectively. In contrast, IL-17 concentrations were significantly higher in the DCC group than in the EPP group at d0 (RD, 95% CI: 1.31–2.90), with mean concentrations of 9.27 ± 6.60 pg/mL in the DCC group and 7.62 ± 2.26 pg/mL in the EPP group.

**Fig. 2:**
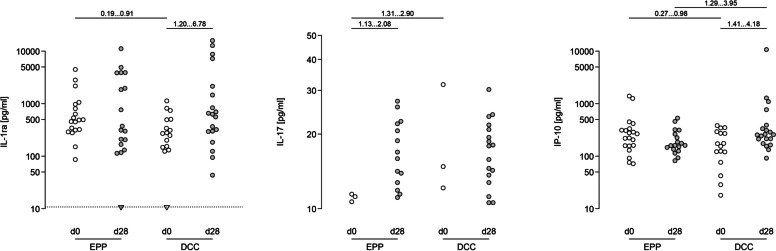
Distribution of cytokines in EPP and DCC groups with statistically significant differences. If applicable, significant relative differences (RD) are given with lower and upper RD of 95% confidence interval (CI). Abbreviations: EPP, extrauterine placental perfusion; DCC, delayed cord clamping

At d28, the adjusted mean cytokine concentrations for infants in the DCC group were significantly higher for IP-10 (RD, 95% CI: 1.29–3.95) than for infants in the EPP group (Fig. 2), with mean concentrations of 877.27 ± 2,336.78 pg/mL in the DCC group and 210.96 ± 123.44 pg/mL in the EPP group. No other significant between-group differences were observed at d28. The distribution of serum levels between the EPP and DCC groups at d0 and d28 for the other cytokines is shown in the supplemental figure (see Additional file 1).

## Discussion

In this randomized comparison of EPP and time-based DCC in VLBW infants, prolonged intact-cord perfusion was not associated with consistent pro-inflammatory shifts across the 27 cytokines measured at birth or at day 28.

Cytokines are thought to play a role in parturition, as several studies report elevated proinflammatory mediators in cord blood. The influence of placental circulation, timing of cord clamping, and the persistence of this inflammatory response remains unclear. Prior studies in term infants report mixed results: Diaz-Castro et al. observed elevated inflammatory parameters after DCC [6], whereas Daskalakis et al. found no differences in TNF-α, IL-1, IL-6, IL-8, or IL-10 between immediate and delayed clamping after 24 h in infants’ blood [8]. Notably, these studies included only healthy term infants, whereas preterm birth is often associated with inflammatory responses [7, 18]. Preterm infants may therefore have a different baseline inflammatory profile from term infants, which limits direct extrapolation form term cohorts to VLBW populations. It is unclear whether preterm birth itself increases cytokine levels, or if other factors, such as infection, cause both preterm birth and elevated cytokine levels [19, 20]. In our cohort, infants with maternal clinical chorioamnionitis or premature rupture of membranes (PROM) did not show elevated inflammatory cytokines. This may reflect the selected study population and the local clinical approach of close monitoring with early cesarean delivery in high-risk pregnancies, potentially limiting exposure to more advanced intrauterine inflammation.

Unlike prior term infant studies, we observed no consistent pro-inflammatory cytokine elevation with prolonged placental circulation in VLBW infants. However, antenatal factors such as corticosteroid exposure and preeclampsia may also have influenced cytokine profiles. Antenatal corticosteroids are generally not thought to significantly alter cord blood concentrations of major pro- or anti-inflammatory cytokines in preterm infants, although some studies suggest a modest attenuation of inflammatory responses in the presence of placental inflammation [21]. This may transiently modify maternal inflammatory markers, but there is no clear evidence that these effects translate into meaningful or sustained changes in fetal or neonatal cytokine levels [22]. In contrast, preeclampsia is more consistently associated with increased cord blood concentrations of IL-6, IL-8, and TNF-α in preterm infants, although the magnitude of this increase is variable and may depend on gestational age and placental pathology [23]. Because antenatal steroid exposure and preeclampsia differed between groups, both variables were included as covariates in the adjusted analyses; however, some confounding cannot be excluded.

Our findings do not support the hypothesis that prolonged cord clamping increases inflammatory cytokines compared with time-based DCC in preterm infants. Importantly, the purpose of the present analysis was not to define normative cytokine ranges, but to explore whether prolonged intact placental circulation is associated with differences in systemic cytokine patterns compared with time-based DCC. The absence of major between-group differences argues against a broad inflammatory effect of EPP under the conditions studied. This negative finding is itself informative because it does not suggest that prolonged intact placental circulation during postnatal stabilization is accompanied by a major systemic cytokine perturbation in this selected cohort.

Among the cytokines showing significant between-group differences, IL-1ra and IP-10 were lower in the DCC group than in the EPP group at d0, whereas IL-17 was higher in the DCC group at d0 and IP-10 was higher in the DCC group at d28. IL-1ra is an anti-inflammatory antagonist of the IL-1 receptor [24]; lower levels in umbilical cord blood with DCC may reflect differences in perinatal anti-inflammatory tone, although no corresponding between-group difference was present at d28. IL-17 is a Th17-related cytokine implicated in neutrophil recruitment [25] and elevated levels are linked to chorioamnionitis. However, higher levels in umbilical cord blood with DCC warrant cautious interpretation given the small number of quantifiable samples and the substantial proportion of values close to or below the assay limits for some analytes. No robust inference regarding biological relevance can therefore be drawn from this isolated difference.

At d28, infants who underwent EPP had lower levels of IP-10 than those with DCC. IP-10 acts as a chemoattractant cytokine that promotes the recruitment of monocytes and other immune cells to the lungs and other organs and has been linked to inflammation-associated morbidities in preterm infants. Plasma IP-10 levels are markedly increased in very preterm infants with late-onset sepsis and necrotizing enterocolitis (NEC) [26]. Similarly, in vitro and in vivo studies demonstrate that IP-10 attracts monocytes and T cells and promotes their adhesion to the endothelium. In pulmonary inflammation models, IP-10 overexpression induces selective accumulation and activation of monocytes and lymphocytes in lung tissue [27]. Lower IP-10 levels at day 28 with EPP may suggest reduced chemoattractant signaling, but causality cannot be inferred from this study. Moreover, because effect estimates were derived from model-based analyses rather than intended as clinical threshold values, the observed between-group differences should be interpreted primarily as relative biological signals rather than as directly clinically actionable concentration changes.

Although these cytokines may contribute to the pathogenesis of inflammatory diseases in preterm infants and EPP might attenuate this response, thereby supporting organ morphogenesis and maturation, the observed cytokine differences in our study did not translate into detectable short-term clinical benefits. The duration of mechanical ventilation, and the incidences of BPD or NEC, were comparable between groups. However, this study was exploratory and hypothesis-generating and was not powered to detect differences in clinical outcomes. Given the relatively low overall incidence of BPD, retinopathy of prematurity (ROP), and NEC among VLBW infants at our center [28], the limited sample size of this study may have further precluded detection of such associations.

Limitations: This study has several limitations. First, the sample size was small, which limits statistical power, particularly for the interpretation of null findings and for analyses of associations between cytokine patterns and clinical outcomes. Second, cytokines were measured at only two predefined time points, at cord clamping and on day 28, which may not capture intermediate or transient inflammatory changes. Third, study inclusion was restricted to cesarean deliveries with prenatal parental informed consent. As a result, acute obstetric situations, particularly emergent cesarean deliveries, could generally not be included because deferred postnatal consent was not permitted by the ethics approval. This likely introduced selection bias toward more stable perinatal conditions and may have contributed to the low rate of clinically diagnosed chorioamnionitis in the present cohort. Conversely, the under-representation of acute inflammatory obstetric situations may also have reduced confounding by overt perinatal inflammation and thereby allowed a clearer characterization of cytokine patterns in this selected cohort of VLBW infants. However, because of the small sample size and selected study population, these data should not be interpreted as reference values for VLBW infants. Fourth, group differences in antenatal steroid exposure and preeclampsia may have influenced cytokine profiles, although analyses were adjusted for these factors. While antenatal steroids are unlikely to have been a major confounder, preeclampsia may represent a more relevant source of bias because of its association with an altered intrauterine inflammatory milieu. Future studies should address these factors in larger multi-center cohorts, ideally with pre-specified stratification or adjustment for major maternal inflammatory conditions. Finally, several cytokines (IL-2, IL-5, IL-7, IL-10, IL-13, IL-15, GM-CSF, VEGF) were below the lower limit of quantification in some samples; but accounted for in statistical analyses, and this may have masked group differences.

## Conclusion

In conclusion, this exploratory analysis suggests that prolonged intact placental perfusion via the EPP approach was not associated with major changes in systemic levels of 27 cytokines measured in VLBW infants at birth or on day 28 compared with time-based DCC. These findings support the biological feasibility of EPP and do not suggest that extended placental circulation is associated with substantial systemic cytokine perturbation under the conditions studied. Given the small sample size and limited number of time points, these preliminary results are hypothesis-generating. Larger studies are warranted to confirm these findings, evaluate intermediate cytokine kinetics, and determine potential clinical implications for neonatal outcomes.

## Supplementary Information

Additional file 1. Distribution of cytokines in EPP and DCC groups without statistical significance. Distribution of cytokines in EPP and DCC groups without statistical significance. If applicable, significant relative differences (RD) are given with lower and upper RD of 95% confidence interval (CI). Abbreviations: CI, confidence interval, EPP, extrauterine placental perfusion; DCC, delayed cord clamping; RD, relative differences.

## Data Availability

The data that support the findings of this study are not openly available due to reasons of sensitivity and are available from the corresponding author upon reasonable request. Data are located in controlled access data storage at University hospital of Cologne.

## Abbreviations

BPD: Bronchopulmonary Dysplasia
CI: Confidence Interval
CRP: C-reactive Protein
CS: Cesarean Section
CPAP: Continuous-positive-airway-pressure
DCC: Delayed Cord Clamping
EPP: Extrauterine Placental Perfusion
ICC: Immediate Cord Clamping
IL: Interleukin
IQR: Inter Quartile Range
IUGR: Intrauterine Growth Restriction
IVH: Intraventricular Hemorrhage
LLOQ: Lower Limit of Quantification
NEC: Necrotizing Enterocolitis
PROM: Premature Rupture of Membranes
RD: Relative Difference
ROP: Retinopathy of Prematurity
SD: Standard Deviation
ULOQ: Upper Limit of Quantification
VLBW: Very Low Birth Weight
