# Supplementary figures and images for: Cytokine patterns in very low birth weight infants under different cord clamping strategies: preliminary EXPLAIN trial data
Source: BMC Pediatr. 2026 Jun 8;26:554. doi: 10.1186/s12887-026-07097-7 (PMC13251227; doi:10.1186/s12887-026-07097-7)

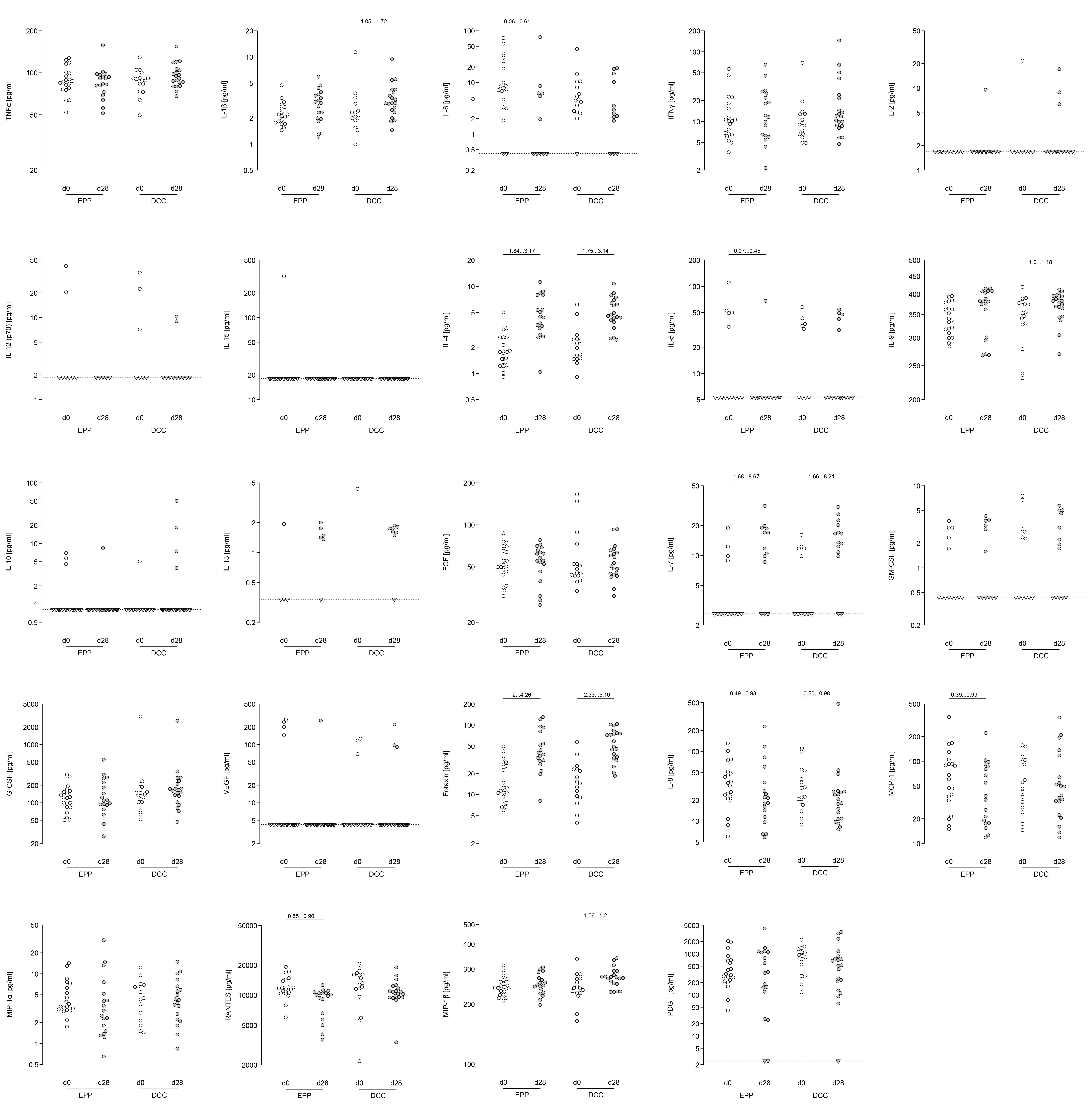

Supplement: Supplementary file 1 — Additional file 1. Distribution of cytokines in EPP and DCC groups without statistical significance. Distribution of cytokines in EPP and DCC groups without statistical significance. If applicable, significant relative differences (RD) are given with lower and upper RD of 95% confidence interval (CI). Abbreviations: CI, confidence interval, EPP, extrauterine placental perfusion; DCC, delayed cord clamping; RD, relative differences. [file 12887_2026_7097_MOESM1_ESM.jpg]
